# Structure and Conservation of Amyloid Spines From the *Candida albicans* Als5 Adhesin

**DOI:** 10.3389/fmolb.2022.926959

**Published:** 2022-07-06

**Authors:** Nimrod Golan, Sergei Schwartz-Perov, Meytal Landau, Peter N. Lipke

**Affiliations:** ^1^ Department of Biology, Technion-Israel Institute of Technology, Haifa, Israel; ^2^ European Molecular Biology Laboratory (EMBL) and Centre for Structural Systems Biology, Hamburg, Germany; ^3^ Biology Department, Brooklyn College of the City University of New York, Brooklyn, NY, United States

**Keywords:** cross-beta, biofilm adhesin, *Candida* pathogenesis, cell wall mannoproteins, functional amyloids

## Abstract

*Candida* Als family adhesins mediate adhesion to biological and abiotic substrates, as well as fungal cell aggregation, fungal-bacterial co-aggregation and biofilm formation. The activity of at least two family members, Als5 and Als1, is dependent on amyloid-like protein aggregation that is initiated by shear force. Each Als adhesin has a ∼300-residue N-terminal Ig-like/invasin region. The following 108-residue, low complexity, threonine-rich (T) domain unfolds under shear force to expose a critical amyloid-forming segment ^322^SNGIVIVATTRTV^334^ at the interface between the Ig-like/invasin domain 2 and the T domain of C*andida albicans* Als5. Amyloid prediction programs identified six potential amyloidogenic sequences in the Ig-like/invasin region and three others in the T domain of *C. albicans* Als5. Peptides derived from four of these sequences formed fibrils that bound thioflavin T, the amyloid indicator dye, and three of these revealed atomic-resolution structures of cross-β spines. These are the first atomic-level structures for fungal adhesins. One of these segments, from the T domain, revealed kinked β-sheets, similarly to LARKS (Low-complexity, Amyloid-like, Reversible, Kinked segments) found in human functional amyloids. Based on the cross-β structures in Als proteins, we use evolutionary arguments to identify functional amyloidogenic sequences in other fungal adhesins, including adhesins from *Candida auris*. Thus, cross-β structures are often involved in fungal pathogenesis and potentially in antifungal therapy.

## Introduction

Potential amyloid-like segments in fungal adhesins are functional in the sense that they mediate microbial adhesion, aggregation, and biofilm formation (Lipke et al., 2017; Tayeb-Fligelman et al., 2017; Evans et al., 2018; Jain and Chapman, 2019). They are amyloid in the sense that they mediate protein assembly into structured β-rich aggregates and fibrils, the same molecular interactions as those in amyloids in neurodegenerative diseases. Amyloid definition is based on the formation of cross-β fibrils composed of tightly mated β-sheets and interdigitating side chains (Shewmaker et al., 2011; Eisenberg and Jucker, 2012; Eisenberg and Sawaya, 2017). Many microbial aggregates and biofilms display these dye-binding properties, and several adhesins form amyloid fibers *in vitro* (Ramsook et al., 2010; Garcia et al., 2011; Lipke et al., 2017)*.* Among the best characterized, gram negative curli are pili composed of proteins assembled through cross-β amyloid-like interactions, and they mediate bacterial adhesion and biofilm formation (Tayeb-Fligelman et al., 2017; Evans et al., 2018; Jain and Chapman, 2019). *In vitro*, anti-amyloid compounds inhibit biofilm formation in bacterial and fungal model systems at concentrations similar to those that inhibit formation of amyloids associated with neurodegenerative diseases and serum amyloidosis (Cegelski et al., 2009; Garcia et al., 2011; Landau et al., 2011; Perov et al., 2019). Thus, the characteristics of microbial functional amyloid mirror those of the better-known pathological amyloids.

Classical amyloids including pathological amyloids in Alzheimer’s disease and serum amyloidoses form fibers that have a characteristic cross-β pattern under X-ray diffraction (Eisenberg and Sawaya, 2017). This pattern shows strong orthogonal reflections of β-strands and β-sheets, with the β-strands orthogonal to the fiber axis and the sheets parallel to the axis. The β-sheets are stacked, and at least one interface between β-sheets is composed of tightly packed interdigitated sidechains, interacting through van der Waals forces and anhydrous H-bonds. This close interdigitation and dry interface give rise to the name “steric zipper,” and the crystalline cross-β structures of short segments are called “amyloid spines” (Eisenberg and Sawaya, 2017).

A second type of homologous crystalline assembly has been recently described: Low-complexity, Amyloid-like, Reversible, Kinked segments (LARKS) (Hughes et al., 2018). These structures also have a cross-β like arrangement, but the β-strands are kinked, the sheet interactions are dependent on smaller interfaces, and the structures are less stable and more evanescent. These LARKs spines are common in low-complexity unstructured regions of proteins (Hughes et al., 2018). Low complexity domains are regions in protein sequences that differ from the composition and complexity of most globular proteins. Such LARKS sequences are involved in RNA-dependent phase separations that influence cellular stress response (Hughes et al., 2018; Hughes et al., 2021); Moreover, LARKS have not been previously reported in adhesins.

Among the best studied fungal adhesins are the paralogs Als1, Als3, and Als5 from *Candida albicans* (Hoyer, 2001; Lipke et al., 2017)*.* Als1 and Als3 are important for fungal virulence in mice, and Als5 can attenuate immune response in the host (Garcia-Sherman et al., 2015; Behrens et al., 2019). Als adhesins are covalently attached to the cell wall glycans and are encoded at 8 loci in the genome (Hoyer et al., 1998; Hoyer, 2001). They are typically 1,200–2,200 residues long (Hoyer, 2001). The N-terminal region is distal to the wall and consists of a 300-residue β-sheet-rich Ig-like/invasin region with two subdomains with Ig-like Greek key folds stabilized by disulfide bonds (Salgado et al., 2011; Lin et al., 2014). In each Als protein, the Ig-like/invasin region is followed by a 108-residue Threonine-rich low-complexity domain (T domain) that is highly conserved in all paralogs and includes a strong amyloidogenic sequence GIVIVA (Otoo et al., 2008). This strong amyloid signature is part of a larger segment, ^322^SNGIVIVATTRTV^334^, which was shown crucial for Als5p amyloid formation (Garcia et al., 2011; Lipke et al., 2017). The entire T region is essential for secretion and processing in yeast (Rauceo et al., 2006). C-terminal to T regions are 5–30 copies of a 36-residue tandem repeat. This region mediates hydrophobic effect interactions with abiotic substrates and with homologous structures. The Als adhesin stalk regions includes the C-terminal 500–1,000 residues and are also of low complexity. These regions are rich in O-glycosylated Ser and Thr residues, as well as in N-glycosylated Asn-Xaa-Ser/Thr motifs. Therefore, the stalks extend up to 200 nm from the surface of the wall. The proteins are synthesized with C-terminal glycosyl phosphatidyl inositol (GPI) anchors, which are subsequently modified to form covalent crosslinks to cell wall glucans (Lipke and Kurjan, 1992; Kapteyn et al., 2000; Roh et al., 2002).

Als5 and other fungal adhesins have characteristics of functional amyloids activated by physical stress. Under extension force applied by liquid flow or atomic force microscopy (AFM) extension, Als adhesin T domains unfold and expose the amyloidogenic sequence GIVIVA from the interface between the Ig/invasin region and the T domain (Lipke et al., 2017). Peptides with this sequence form amyloid fibers, as do soluble forms of Als5, but peptides of fragments with a non-amyloid substitution GINIVA do not form amyloids (Otoo et al., 2008; Ramsook et al., 2010). When anchored to the cell surface, exposure of the GIVIVA sequence leads to formation of high avidity patches of adhesins on the cell surface (Alsteens et al., 2010). These patches consist of adhesins aggregated through amyloid-like bonds, and as a result, cells expressing these adhesins bind amyloidophilic dyes strongly (nM concentrations of thioflavins) (Garcia et al., 2011). Furthermore, aggregates of cells expressing these adhesins are birefringent in polarized light, a phenomenon not seen in the controls (Otoo et al., 2008; Ramsook et al., 2010). Recent evidence also shows that cell-to-cell binding is mediated by amyloid-like interactions between cells for Als5 and Als1 (Dehullu et al., 2019a; Dehullu et al., 2019b; Ho et al., 2019). Thus, formation of amyloid-like interactions mediates strong bonding between fungal cells, especially in biofilms formed under flow. Furthermore, amyloid-like structures coat the surface of fungi in fatal invasive fungal infections caused by many species. In superficial and invasive candidiasis, Als proteins are major components of these surface amyloid-like structures (Garcia-Sherman et al., 2014; Garcia-Sherman et al., 2015; Klotz et al., 2016). The fungal surface amyloid attenuates macrophage response to the invading fungi (Behrens et al., 2019). Thus, extensive amyloid-like interactions are common on adhering fungi and in fungal biofilms and can influence host response.

Amyloidogenic sequences in fungal adhesins have been widely predicted, and force-dependent formation of amyloid-like surface nanodomains has been demonstrated in several cases (Chan and Lipke, 2014; Lipke et al., 2017; Lipke, 2018; Bouyx et al., 2021). However, no atomic-level structures of amyloid spines or steric zippers from this type of protein have been reported. The adhesin Als5 conforms to the general model in its sequence, with an Ig/invasin region (residues 20–325), a 108-residue T region, six tandem repeats, and a 750-residue unstructured stalk. The tandem repeats and stalk are low complexity sequences, rich in serine and threonine residues, and are glycosylated on up to a third of all residues (Rauceo et al., 2004; Lipke, 2018). Such high glycosylation is not compatible with amyloid formation through cross-β structures, and therefore we restricted our analysis to the Ig-like/invasin and T regions. We have screened the Als5 Ig-like/invasin and T domains for predicted amyloidogenic sequences and determined structural characteristics of several of these. We have also analyzed the conservation and potential *in vivo* roles of these sequence segments in Als5 and homologous proteins.

## Materials and Methods

### Peptides and Reagents

Peptide segments ^156^NTVTFN^161^, ^168^SIAVNF^173^, ^196^IATLYV^201^, ^324^GIVIVA^329^ and ^369^TSYVGV^374^ from Als5 (UniProt accession number Q5A8T7) were synthesized at >98% purity and purchased from GL Biochem (Shanghai) Ltd. The peptides were synthesized with unmodified termini for crystallography or with fully capped termini (acetylated in the N-terminus and amidated in the C structural terminus) for fibrillation assays. Thioflavin T (ThT) was purchased from Sigma-Aldrich. Dimethyl sulfoxide (DMSO) was purchased from Merck. Ultra-pure water was purchased from Biological Industries.

### Computational Prediction of Amyloid Spine Segments

Amyloidogenic propensities of Als5 segments were predicted using combined information from several computational methods, including TANGO (Fernandez-Escamilla et al., 2004), AmylPred 2 (Tsolis et al., 2013) and FiSH Amyloid (Gasior and Kotulska, 2014). TANGO and FiSH Amyloid were used in their default settings, and AmylPred 2 was used with the default consensus option of only 4 of 9 predictors (TANGO and Amyloid Mutants were omitted from its prediction). We used TANGO, AmylPred 2, and FiSH Amyloid because they were the most sensitive and consistent predictors with quantifiable scores. The spine segments were defined as short segments of 6 residues, detected by at least two methods out of three at the epicenter of the predicted amyloidogenic area in the sequence (like in the case of ^369^TSYVGV^374^). Only in the case of the segment ^322^SNGIVIVATTRTV^334^, which is known to be critical to fibrillation of the Als5 protein (Garcia et al., 2011), we worked with both its full length and a shorter version of 6 residues.

### Modelling Als5 Structure

The three-dimensional (3D) model of the entire Als5 sequence from *Candida albicans*, taken directly from the UniProt accession number Q5A8T7 was generated by AlphaFold v2.1.0 pipeline (Jumper et al., 2021; Varadi et al., 2022). Using Chimera (Goddard et al., 2007), we focused only on residues 20–433 which include the Ig-like/invasin and T domains. The per-residue confidence score (pLDDT) of the Ig-like domain ranged above 90 indicating a reliable prediction (Mariani et al., 2013). For the T domain, the per-residue confidence score ranged between 70 and 90 indicating lower confidence in the prediction.

### Calculation of Evolutionary Conservation Using the ConSurf/CenSeq Webserver

We used ConSurf (Landau et al., 2005; Ashkenazy et al., 2016) with default settings to examine the evolutionary conservation patterns of *Candida albicans* Als5 residues 20–433 which include the Ig-like and T domains. The calculation was run using ConSeq (Berezin et al., 2004) (no structure was provided). One hundred and twenty-one unique sequences of homologs with 35%–95% sequence identity were retrieved from the UniRef90 database. Homologs with large gaps (more than 5 residues) in their alignment were filtered using Jalview2 multiple sequence alignment editing program (Waterhouse et al., 2009). The alignment of the remaining 71 homologs was used for the calculations presented here. The alignment is accessible at https://drive.google.com/file/d/1jWLOldYEx5aZz5VnrlnYv7lvYcN6DgkO/view?usp=sharing. The alignment of the seven orthologs used to generate the information presented in Figure 5 is accessible at https://drive.google.com/file/d/1SFTarMIUj762evoOPkWXi-S56jTezG45/view?usp=sharing.

### Amyloidogenic Propensity Correlated to the Evolutionary Conservation of the Segments

Comparison between the segments ^156^NTVTFN^161^, ^196^IATLYV^201^, ^369^TSYVGV^374^, and ^322^SNGIVIVATTRTV^334^ evolutionary conservation was done by averaging the amino acid conservation scores attained from the ConSurf results for each segment. The segments ^177^TVDQSG^182^ and ^238^VNDWNH^243^ were taken as controls due to their lack of amyloidogenic propensity and their different conservation values, namely low and high respectively. The 71 retrieved homologs were ranked by their sequence similarity to *C. albicans* Als5, with the lowest *e*-value corresponding to the closer homologs of *C. albicans* Als5. Out of the 71 homologs we selected one along every five ranked homologs (in descending order of similarity to *C. albicans* Als5) for a total of fourteen sequences representing diverse sequences in the multiple sequence alignment. We then calculated the amyloidogenic propensity for each of the fourteen homologs using TANGO (Fernandez-Escamilla et al., 2004), AmylPred 2 (Tsolis et al., 2013), and FiSH Amyloid (Gasior and Kotulska, 2014) (Figures 4B, 5). The multiple sequence alignment was used, via Jalview2 (Waterhouse et al., 2009), to identify equivalent regions the segments of *C. albicans* discussed here. We then calculated the average predicted amyloidogenic propensity values in these corresponding sequences in each of the 14 homologs. TANGO averages were calculated by dividing the sum of each residue’s amyloidogenic propensity by its length. AmylPred 2 averages were calculated by assigning to each residue of the segments either a Boolean value of zero or one (based on consensus recognition of the method) and calculating the average similarly to TANGO averages. Using the default sliding window settings of the method, FiSH Amyloid averages were calculated by dividing the sum of the values assigned to all residues covering the segment by its length.

For the calculations of the amyloidogenic propensity of the entire Ig-like/invasin and T domains, we selected seven orthologs (homologs with a common ancestral parent in different species). Six of those were chosen along the ranked 71 homologs in gaps of 10 (starting from *C. dubliniensis* orthologs located five places away from the original *C. albicans* Als5). To these six, we added the emerging pathogen *Candida auris*, which was not a part of the homologs collected by the ConSurf webserver due to low sequence identity to the *C. albicans* Als5p (Willaert, 2018; Willaert et al., 2021). The multiple sequence alignment of the 71 homologs along with the *C. auris* sequence was obtained by reproducing the ConSurf search with similar parameters but lower sequence identity of 25%–95%, and then filtering out the irrelevant homologs using Jalview2.

### Thioflavin T Kinetic Assays

Thioflavin T (ThT) is a commonly used for identifying and investigating the formation of amyloid fibrils *in vivo* and *in vitro*. In the presence of ThT, fibrillation curves commonly show delayed nucleation followed by rapid aggregation. Fibrillation kinetics of the peptides ^156^NTVTFN^161^, ^196^IATLYV^201^, ^369^TSYVGV^374^, and ^322^SNGIVIVATTRTV^334^ were monitored using ThT. All of the above peptides were synthesized with fully capped termini (acetylated in the N-terminus and amidated in the C structural terminus) to mimic its chemical nature in the full-length protein, and were dissolved to 10 mM in DMSO. Each of the freshly dissolved peptides was mixed with 50 mM Tris-HCl buffer with pH 7.3 and with 2 mM filtered ThT stock (made in ultra-pure water) to reach final concentrations of 300 μM of peptide and 20 μM ThT in the final volume of 100 μl reaction. The reaction mixture was carried out in a black 96-well flat-bottom plate (Greiner bio-one) covered with a thermal seal film (EXCEL scientific) and incubated in a plate reader (CLARIOstar, BMG Labtech), at 37°C, with orbital shaking at 300 rpm, for 30 s before each measurement. ThT fluorescence was recorded every 2 minutes for a total time of ∼48 h (we show only 5 h of the measurements) using an excitation of 438 ± 20 nm and an emission of 490 ± 20 nm. The measurements were conducted in triplicates, and the entire experiment was repeated at least three times.

### Transmission Electron Microscopy

TEM was used to visualize fibrils. Samples for TEM were taken directly from the ThT kinetic assay plate, which was left to incubate at 37**°**C for 2 days with 300 rpm shaking in the plate reader. The TEM grids were prepared by applying 5 µl samples of each 300 μM sample of peptide on 400 mesh copper grids with support films of Formvar/Carbon (Ted Pella), that were charged by high-voltage, alternating current glow-discharge (PELCO easiGlow, Ted Pella) immediately before use. Samples were allowed to adhere for 1 min followed by negative staining with 1% uranyl acetate for 1 min. Micrographs were recorded using a FEI Tecnai G2 T20 S-Twin transmission electron microscope at an accelerating voltage of 200 KeV at the MIKA electron microscopy center of the Department of Material Science & Engineering at the Technion.

### Fiber X-Ray Diffraction of the SNGIVIVATTRTV Segment From Als5

The peptide SNGIVIVATTRTV was dissolved to 10 mg/ml in ultra-pure water. A few microliters were applied between two sealed glass capillaries until completely dried. X-ray diffraction of the samples was collected at the micro-focused beam P14 at the high brilliance 3rd Generation Synchrotron Radiation Source at DESY: PETRA III, Hamburg, Germany.

### Crystallization of Als5 Segments

Peptides synthesized with free (unmodified) termini were used for crystallization experiments to facilitate crystal contacts. All peptides were dissolved to 10 mM in ultrapure water if possible, and in DMSO in case they were water-insoluble. ^196^IATLYV^201^ was dissolved in 95% DMSO, ^156^NTVTFN^161 168^SIAVNF^173^ and ^324^GIVIVA^329^ were dissolved in 100% DMSO. ^369^TSYVGV^374^ was dissolved in 100% ultrapure water. Peptide solution drops (100 nl) were dispensed onto crystallization screening plates, using the Mosquito automated liquid dispensing robot (TTP Labtech, United Kingdom) located at the Technion Center for Structural Biology (TCSB). Crystallization using the hanging drop method, was performed in 96-well plates, with 100 µl solution in each well. The drop volumes were 150–300 nl. All plates were incubated in a Rock imager 1,000 robot (Formulatrix), at 293K. Micro-crystals grew after few days and were mounted on glass needles glued to brass pins. No cryogenic protection was used. Crystals were kept at room temperature prior to data collection. Structures were obtained from drops that were a mixture of the following peptide and reservoir solutions: ^196^IATLYV^201^: 10 mM IATLYV, 0.1 M tri-Sodium citrate pH 5.6 and 1.0 M Ammonium phosphate; ^156^NTVTFN^161^: 10mM NTVTFN, 0.1 M HEPES pH 7.5, 0.8 M NaH_2_PO_4_, 0.8 M KH_2_PO_4_; ^369^TSYVGV^374^: 10 mM TSYVGV, 0.2 M Sodium chloride, 0.1 M Sodium acetate pH 4.6, 30% (v/v) 2-Methyl-2,4-pentanediol (MPD).

### Structure Determination and Refinement

X-ray diffraction data were collected at 100K, using 5° oscillation. The X-ray diffraction data were collected at the micro-focus beamline ID23-EH2 of the European Synchrotron Radiation Facility (ESRF) in Grenoble, France; wavelength of data collection was 0.8729 Å. Data indexation, integration and scaling were performed using XDS/XSCALE (Kabsch, 2010). Molecular replacement solutions for all segments were obtained using the program Phaser within the CCP4 suite (Murshudov et al., 1997; McCoy et al., 2007; Winn et al., 2011). The search models consisted of geometrically idealized β-strands. Crystallographic refinements were performed with the program Refmac5 (Murshudov et al., 1997). Model building was performed with Coot (Emsley et al., 2010) and illustrated with Chimera (Goddard et al., 2007). There were no residues that fell in the disallowed region of the Ramachandran plot. Crystallographic statistics are listed in Table 1.

**TABLE 1:** Data collection and refinement statistics.

PDB ID	Als5^196^IATLYV^201^	Als5^156^NTVTFN^161^	Als5^369^TSYVGV^374^
	6RHB	6RHA	6RHD
Beamline	ESRF ID23_2	ESRF ID23_2	ESRF ID23_2
Date	7 September 2015	10 May 2015	11 December 2015
Data collection			
Space group	P 21 21 21	P 1 21 1	P 1 21 1
Cell dimensions			
a, b, c (Å)	9.49 17.87 22.42	4.87 39.81 20.26	11.77 9.36 16.79
α, β, γ (°)	90.0 90.0 90.0	90.0 90.0 90.0	90.0 95.1 90.0
Wavelength (Å)	0.8729	0.8729	0.8729
Resolution (Å)	22.4–1.26 (1.32–1.26)	39.8–1.60 (1.69–1.60)	16.73–1.20 (1.25–1.20)
R-factor observed (%)	24.6 (75.7)	27.0 (100.5)	20.0 (85.5)
^a^ *R*_meas_ (%)	25.8 (79.5)	28.0 (108.4)	20.4 (90.0)
*I*/σ*I*	6.8 (2.9)	7.3 (2.2)	13.7 (2.5)
Test set size [%], selection	10, random	10, random	10, random
Total reflections	12,416 (984)	14,151 (893)	31,912 (1,290)
Unique reflections	1,076 (90)	991 (126)	1,178 (139)
Completeness (%)	91.1 (58.8)	98.2 (90.0)	96.2 (97.9)
Redundancy	11.5 (10.9)	14.3 (7.1)	27.1 (9.3)
^b^CC1/2 (%)	99.5 (97.1)	97.5 (72.7)	99.9 (84.2)
Refinement			
Resolution (Å)	13.97–1.26 (1.41–1.26)	19.91–1.60 (1.64–1.60)	16.73–1.20 (1.23–1.20)
Completeness (%)	91.11 (76.6)	97.9 (77.9)	96.2 (98.8)
^c^ No. reflections	968 (218)	891 (54)	1,059 (75)
^d^ *R*_work_ (%)	9.97 (17.5)	19.8 (29.0)	10.6 (21.7)
*R*_free_ (%)	10.39 (24.1)	21.3 (22.1)	12.6 (22.3)
No. atoms	49	103	51
Protein	48	Chain A: 49 Chain B: 49	44
Water	1	5	7
B-factors			
Protein	4.8	Chain A: 11.1 Chain B: 12.4	7.25
Water	7.2	19.4	24.1
R.m.s. deviations			
Bond lengths (Å)	0.007	0.014	0.011
Bond angles (°)	1.560	1.634	1.545
Clash score Kabsch (2010)	0.00	0.00	0.00
Molprobity score Kabsch (2010)	0.5	0.5	0.5
Molprobity percentile Kabsch (2010)	100th percentile	100th percentile	100th percentile
Number of xtals used for scaling	One crystal, one data set	Two crystals, one data set from each	One crystal, six data sets

Values in parentheses are for highest-resolution shell.

a R-meas is a redundancy-independent R-factor defined in Diederichs and Karplus (1997).

b CC_1/2_ is percentage of correlation between intensities from random half-datasets (Karplus and Diederichs, 2012).

c Number of reflections corresponds to the working set.

d Rwork corresponds to working set.

### Calculations of Structural Properties

The Lawrence and Colman’s shape complementarity index (Lawrence and Colman, 1993) was used to calculate the shape complementarity between pairs of sheets forming the dry interface (Supplementary Table S1). The buried surface area was calculated with Chimera (UCSF) (Goddard et al., 2007), with a default probe radius and vertex density of 1.4 Å and 2.0/Å^2^, respectively. The number of solvent accessible buried surface areas was calculated as the average area buried of one strand within two β-sheets (total area buried from both sides is therefore double the reported number in Supplementary Table S1.

### Computational Prediction of Als5 Ig-Like and T-Domains Multimeric Assembly

A prediction of the multimeric assembly of Als5 Ig-like and T-domains was done with AlphaFoldv2 Advanced pipeline (modified AlphaFold v2.1.0 Multimer pipeline) (Jumper et al., 2021) (Richard Evans et al. bioRxiv 2021). The pipeline was executed *via* the Google Colab service available online to each AlphaFold program (AlphaFold v2.1.0—https://colab.research.google.com/github/deepmind/alphafold/blob/main/notebooks/AlphaFold.ipynb, AlphaFold v2.1.0 advanced—https://colab.research.google.com/github/sokrypton/ColabFold/blob/main/beta/AlphaFold2_advanced.ipynb#scrollTo=pc5-mbsX9PZC). The prediction was based on the sequence of Als5 20–433 residues (UniProt accession number Q5A8T7). A total of five homodimers of Als5 20–433 were retrieved and were ranked based on their per-residue confidence score. Models of homodimers were illustrated, colored, and analyzed using UCSF Chimera (Goddard et al., 2007).

## Results

### Potential Amyloid Core Sequences in *Candida albicans* Als5

**FIGURE 1:**
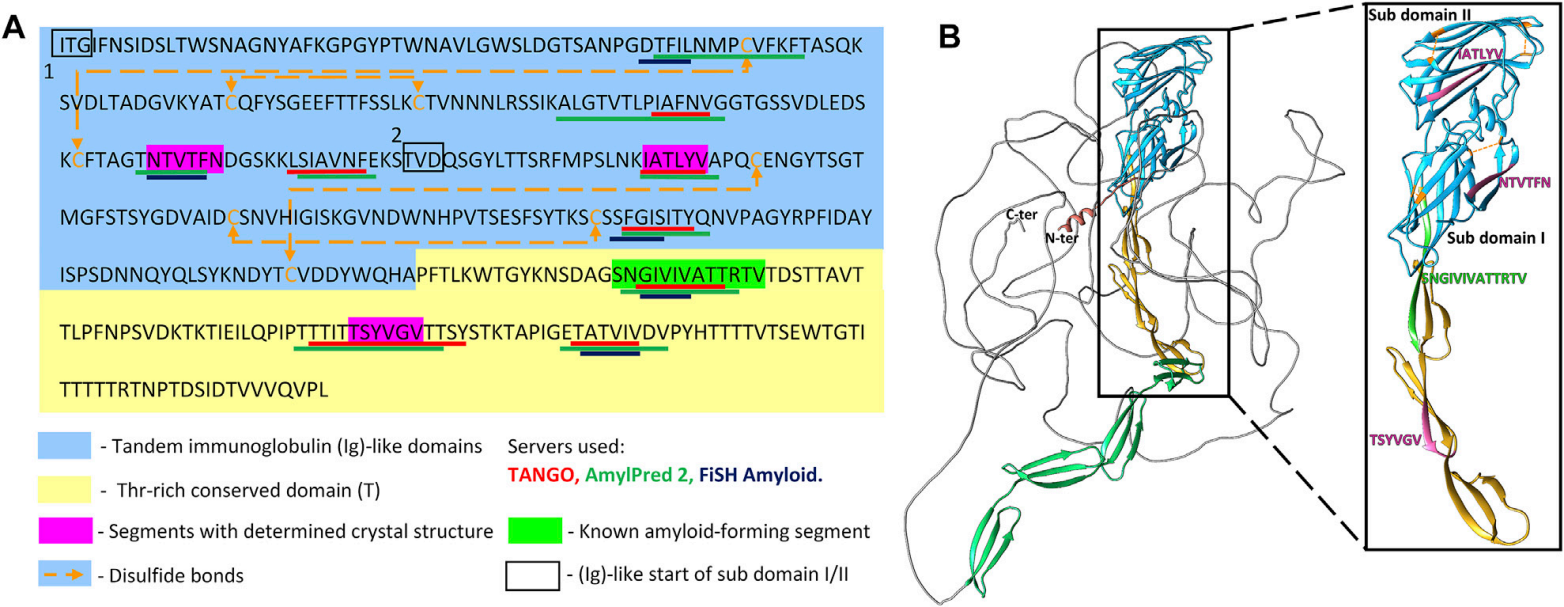
Predicted amyloidogenic segments within Als5 sequence and modeled structure **(A)** Annotated N-terminal residues 20–433 of the *Candida albicans* Als5 protein containing the Ig-like/invasin and T regions, indicated by blue and yellow backgrounds, respectively. Segments with strong amyloidogenic predictions are denoted with colored bars under the sequence: TANGO (Fernandez-Escamilla et al., 2004) in red, AmylPred 2 (Tsolis et al., 2013) in green, and FiSH Amyloid in blue (Gasior and Kotulska, 2014). Segments for which the crystal structure was determined are marked magenta, and the known amyloid segment ^303^SNGIVIVATTRTV^315^ is marked green. Disulfide bonds are illustrated by orange dashed arrow lines. **(B)** A three-dimensional (3D) model of the Als5 predicted by AlphaFold Monomer v2.0 (Jumper et al., 2021; Varadi et al., 2022). Alphafoldv2 predicted a folded structure for the Ig/T domains (blue and yellow) and four subdomains of the TR region (green), but not for the rest two tandem repeats and the C-terminal 850-residue glycosylated stalk region (grey line). Right inset is an enlarged image of residues 20–433 with the two Ig-fold subdomains indicated.

Sequence- and structure-based amyloid predictors use a variety of criteria including solubility and physicochemical properties, geometry, homology, and secondary structure propensity to predict occurrence of amyloidogenic core regions that can form cross-β aggregates and steric zippers. Accordingly, we scanned the primary structure of Als5 Ig/invasin and T domains (residues 20–433) with three amyloid prediction programs. TANGO has been useful for predictions in Als adhesins, is a thermodynamics state-based predictor (Fernandez-Escamilla et al., 2004; Lipke et al., 2017). AmylPred 2 is a consensus method based on eleven predictors (Tsolis et al., 2013). Lastly, FiSH Amyloid detects co-occurrence patterns in sequence data based on machine-learning techniques (Gasior and Kotulska, 2014). Nine segments scored above threshold in at least two of the three predictors, marked on the Als5 sequence in Figure 1A. Six segments are in the Ig-like/invasin region: ^65^DTFILN^70^, ^132^IAFNVG^137^, ^156^NTVTFN^161^, ^168^SIAVNF^173^, ^196^IATLYV^201^, and ^259^FGISIT^264^, and three sequences are in the T domain: ^324^GIVIVA^329^, a section from the longer segment ^322^SNGIVIVATTRTV^334^ at the Ig/T domain interface, ^369^TSYVGV^374^, and ^388^TATVIV^393^. These segments were mapped onto an AlphaFold Monomer v2.0 model of Als5p (Jumper et al., 2021; Varadi et al., 2022) (Figure 1). AlphaFoldv2 predicted a folded structure for the Ig/T domains and the first four tandem repeats regions. As expected, there was no structural prediction for the highly glycosylated low complexity C-terminal stalk region, which is unstructured and in extended conformation *in vivo* (Lipke et al., 2017).

### Structures of Amyloidogenic Spines From Als5

**FIGURE 2:**
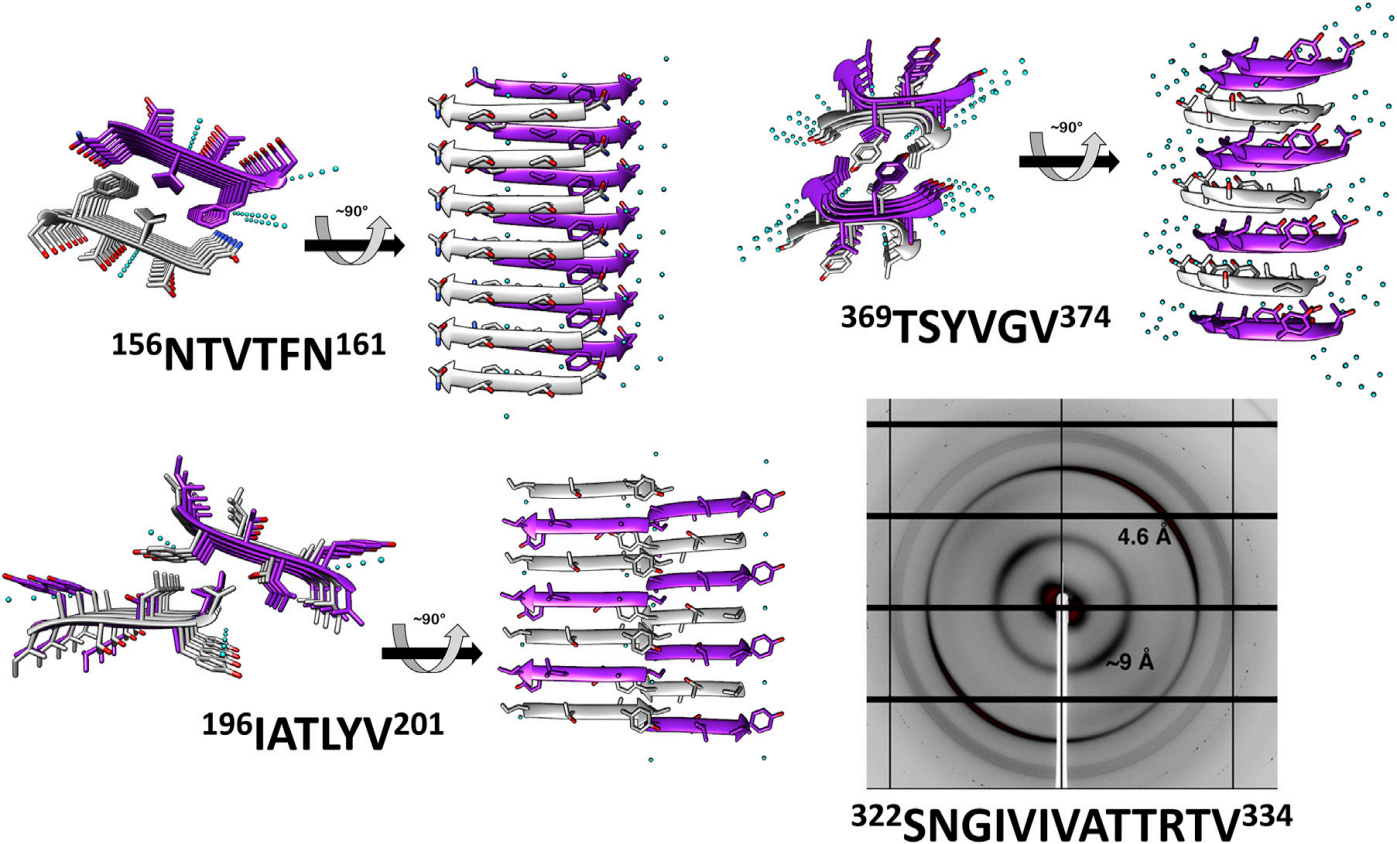
Crystal structures and X-ray fiber diffraction of Als5 amyloidogenic segments. High-resolution crystal structure of three predicted amyloidogenic segments of Als5 and the known amyloid segment ^322^SNGIVIVATTRTV^334^. The left panel of each structure is viewed down the fibril axis, with β-strands shown as ribbons and residues as sticks, while the right panel is viewed perpendicular to the fibril axis displaying β-strands running horizontally. Fibrils contain thousands of layers of β-strands, but only seven layers are shown here. Segment backbones are colored either gray or purple; heteroatoms are colored according to their atom type (nitrogen in blue, oxygen in red) and water molecules in cyan. The X-ray fiber diffraction pattern with major reflections labeled showing a cross-β pattern with orthogonal reflections at 4.6 Å and ∼9 Å spacings. Some reflections originating from ice also appear.

We determined the atomic structures of ^156^NTVTFN^161^, ^196^IATLYV^201^ and ^369^TSYVGV^374^ segments (PDB IDs 6RHA, 6RHB, and 6RHD, respectively; Figure 2, Table 1, and Supplementary Table S1). The locations of the amyloid segments on the AlphaFoldv2 predicted structure of Als5 are shown in Figure 1B. The crystal structure of the ^156^NTVTFN^161^ spine segment from the Ig-like domain formed a canonical steric-zipper structure of tightly mated parallel β-sheets with a dry interface. The ^196^IATLYV^201^ spine segment, also from the Ig-like subdomain 2, revealed a partial mating between antiparallel β-sheets. The ^369^TSYVGV^374^ spine segment from the T domain of Als5 exhibited an atypical amyloid structure, with antiparallel β-sheets and kinked β-strands (Figure 2 and Supplementary Figure S1).

**FIGURE 3:**
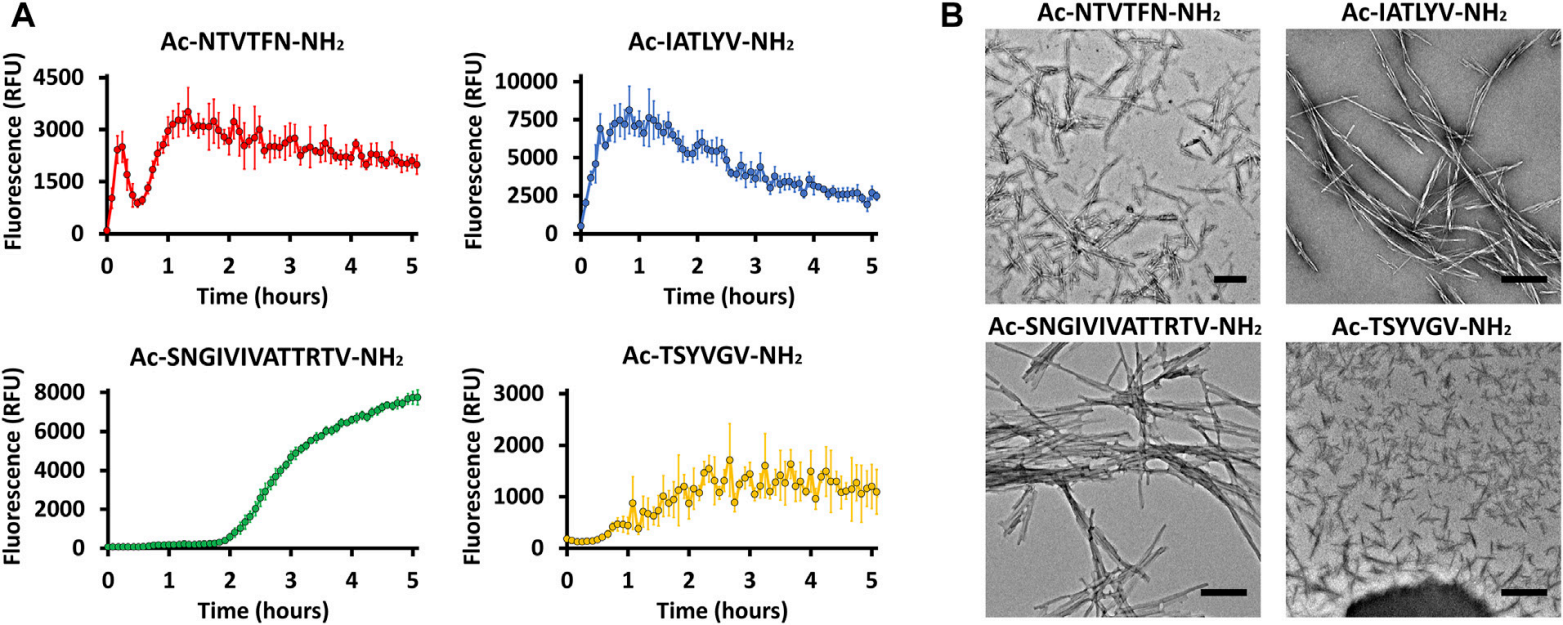
Predicted segments exhibiting amyloidogenic properties **(A)** Segments for which the crystal structure was determined and ^322^SNGIVIVATTRTV^334^ show fibrillation when incubated with ThT. Measurements were conducted in 50 mM Tris-HCl buffer, 20 mM ThT, and 300 µM of peptide. The graph shows the average of three technical repeats, with error bars representing standard deviations. **(B)** Electron micrographs of the four segments show fibril formation after 48 h of incubation. Scale bar represents 300 nm.

These peptides induced development of thioflavin-T (ThT) fluorescence typical of amyloid aggregation (Figure 3A) and formed amyloid-like fibrils visualized by transmission electron microscopy (Figure 3B). The peptides ^156^NTVTFN^161^ and ^196^IATLYV^201^ each formed abundant and long fibrils, although without the typical lag associated with amyloid formation, possibly due to presence of seeds in the original sample. ^369^TSYVGV^374^ formed abundant short plump bundled fibrils. The longer peptide ^322^SNGIVIVATTRTV^334^ segment, known to be critical for biological activity of the adhesin and crucial for fibrillation of soluble forms of Als5 protein, formed long and relatively straight fibrils that also induced ThT fluorescence (Lipke et al., 2017). This sequence contains a strongly predicted amyloid core sequence ^324^GIVIVA^329^. Although we did not successfully crystallize either the 6-residue or the 13-residue segments, the X-ray fiber diffraction of ^322^SNGIVIVATTRTV^334^ showed a canonical amyloid cross-β pattern with orthogonal reflection arches at 4.6 and ∼9 Å (Figure 2).

Because steric-zipper fibrils are unusual in that pairs of β-sheets mate more closely than the adjoining surfaces in other protein complexes, quantitative measures of amyloid stability are based on solvent-accessible surface area buried at the interface between the mating sheets, and shape complementarity indicating on the closeness of fit of two protein surfaces (Tayeb-Fligelman and Landau, 2017). The shape complementarity, inter-strand distance, and solvent-exposed surface area buried were calculated for Als5 spine structures and compared to the NNQQNY segment from yeast prion Sup35 steric zipper (PDB ID 1YJO) (Lawrence and Colman, 1993). The NNQQNY structure was chosen for this comparison as it shows one of the highest values of shape complementarity and surface area buried among steric zipper structures (Eisenberg and Sawaya, 2017). The shape complementarity was similar for all Als5 spines segment, all lower than prion NNQQYY (Supplementary Table S1). However, the T domain ^369^TSYVGV^374^ spine segment, displaying kinked β-strands, showed a significantly lower solvent-exposed surface area buried compared to the other structures (Supplementary Table S1), indicating a less stable structure that might also suggest reversible fibril formation.

The ^156^NTVTFN^161^ and ^196^IATLYV^201^ segments in the Ig-like/invasin region are each close to a disulfide bonded Cys residue (Figure 1). Therefore, it is likely that these sequences would not have the conformational freedom to lead to solvent exposure or interaction necessary for amyloid formation. In contrast, the amyloid-forming segments in the T region, which is conformationally variable *in vivo*, are not constrained by disulfides or other structured regions. Thus, the amyloid propensity in this region is likely to be expressed *in vivo*, as demonstrated before for ^322^SNGIVIVATTRTV^334^ (Lipke et al., 2017).

### Patterns of Evolutionary Conservation and Amyloidogenic Propensities of Amyloid Segments in Fungal Adhesins

Evolution can shift and alter traits in all biological systems. Selection pressure will work to conserve traits that possess an important beneficial function. It is also true for functional amyloids, where purifying selection acts to conserve the amyloidogenic potential, amino acid sequence, and accessible conformation; however, the amyloidogenic potential and amino acid sequence are not always conserved equally. Occasionally, amino acid sequences can change considerably without greatly affecting their amyloidogenic potential (Maji et al., 2009). In light of this difference, we have examined the evolutionary conservation patterns of the amyloidogenic segments correlated with the trajectory of the change in amyloidogenic potential from close to more distant homologs of *C. albicans* Als5.

**FIGURE 4:**
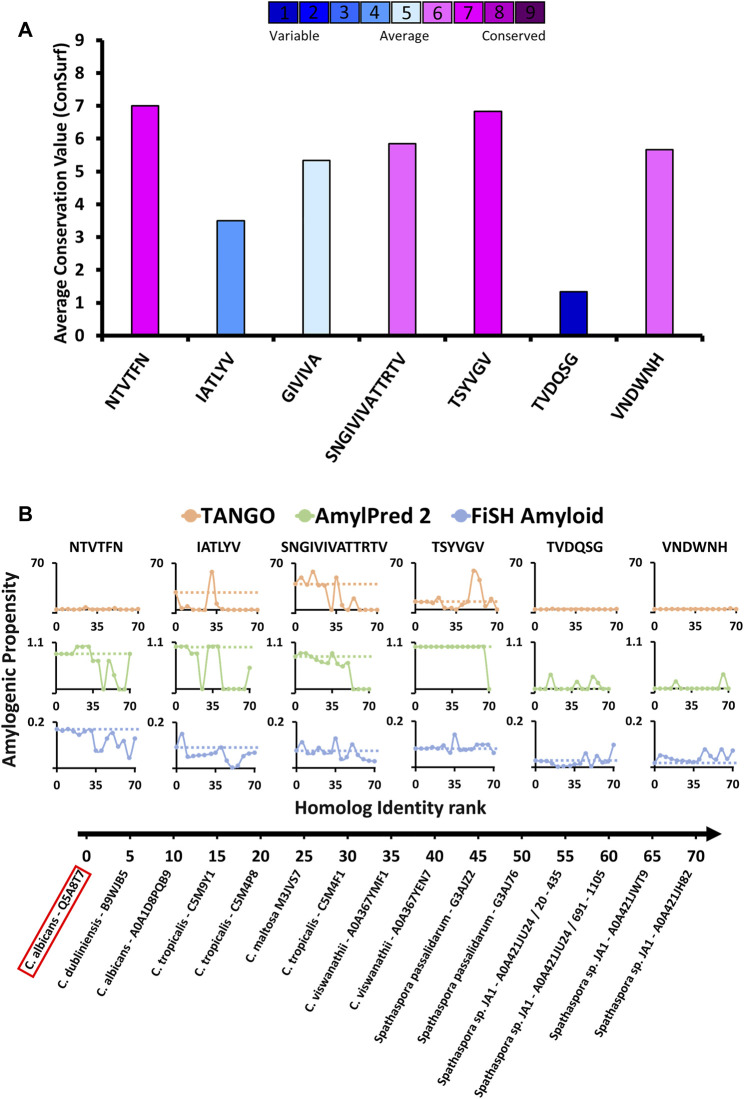
Amyloidogenic propensity and the evolutionary conservation of *C. albicans* Als5 segments. **(A)** Evolutionary conservation of Als5 segments. The graph displays evolutionary conservation values for seven segments (three segments with resolved crystal structures, known amyloid spine ^322^SNGIVIVATTRTV^334^, and two control segments) from *C. albicans* Als5. These values were calculated based on a multiple sequence alignment of 71 homologs of Als5 generated using the ConSurf webserver (Berezin et al., 2004; Landau et al., 2005; Ashkenazy et al., 2016). The columns’ colors correspond to the average evolutionary conservation score (1 being the least conserved and 9 being the most conserved). **(B)** The amyloidogenic propensities of the above seven segments in Als5 orthologs (homologs from different species). Fourteen representative orthologs of Als5p were selected to represent a diverse group (as described in the Method section). The orthologs are plotted on the *X*-axis by their sequence similarity to *C. albicans* (evaluated based on ConSurf *e*-value parameter) from most to least similar homolog. The *Y*-axis shows the average amyloidogenic propensity of each segment in the fourteen orthologs. The amyloidogenic values were calculated using three servers and displayed using different colors: TANGO (orange) (Fernandez-Escamilla et al., 2004), AmylPred2 (green) (Tsolis et al., 2013), FiSH Amyloid (blue) (Gasior and Kotulska, 2014). The different methods have a different score range, thus in each graph, a horizontal dashed line indicates the amyloidogenic value of the relevant segment from *C. albicans* Als5.

To evaluate the conservation of the amyloidogenic segments in protein homologous to Als5 we used the ConSurf webserver (Berezin et al., 2004; Ashkenazy et al., 2016). First, we searched for homologs for Als5 residues 20–433 which include the Ig-like/invasin and T domains. Seventy-one unique sequences of homologs with 35%–95% sequence identity were retrieved from the UniProt (UniRef90) database. We excluded homologs with large gaps in the multiple sequence alignment. The segments ^156^NTVTFN^161^, ^196^IATLYV^201^, ^322^SNGIVIVATTRTV^334^, ^324^GIVIVA^329^ and ^369^TSYVGV^374^ showed diverse pattern of sequence conservation across the Als5 homologs. In the Ig-like/invasin region, the sequence ^156^NTVTFN^161^ was remarkably conserved across the homologs (Figure 4A), with five strongly conserved positions (Supplementary Figure S2), implying a conserved functional or structural role. In contrast, ^196^IATLYV^201^ was not well conserved and showed low average conservation value with four highly variable positions in its sequence. In the T domain, the ^322^SNGIVIVATTRTV^334^ region showed intermediate to above-average conservation. This observation is in agreement with previous reports showing that the functional amyloid-forming sequence ^322^SNGIVIVA^329^ is strongly conserved within the *C. albicans ALS* gene family (Otoo et al., 2008; Ho et al., 2019). The LARKS-like sequence ^369^TSYVGV^374^ also showed intermediate average conservation value with three highly conserved positions, with highest value at Gly^373^.

We also evaluated the conservation of amyloidogenic potential within the Als5p sequence homologs (Figure 4B). The spine-forming segments ^156^NTVTFN^161^, ^196^IATLYV^201^, ^322^SNGIVIVATTRTV^334^, and ^369^TSYVGV^374^ presented a wide range of differences in amyloidogenic potential calculated by three predictors: TANGO, AmylPred 2, and FiSH Amyloid. For further analyses, we selected as controls two segments with a predicted low amyloidogenic propensity, ^177^TVDQSG^182^ and ^238^VNDWNH^243^, showing low and intermediate evolutionary conservation, respectively. The ^322^SNGIVIVATTRTV^334^ segment already implicated in Als5 function (Lipke et al., 2017) showed a high amyloidogenic propensity in close homologs, which declined in more distant homologs. The LARKS-like sequence ^369^TSYVGV^374^, which is relatively conserved (Figure 4A), showed the highest stability in maintaining amyloidogenic potential across homologs according to Amylpred2 and FiSH amyloid, with more fluctuations in propensity towards more distant homologs (Figure 4B). In contrast, the Ig-like/invasin region segments ^156^NTVTFN^161^ and ^196^IATLYV^201^ show a less clear pattern of amyloidogenic propensity across homologs, similar to the control sequences ^177^TVDQSG^182^ and ^238^VNDWNH^243^. Control sequence ^177^TVDQSG^182^ showed high fluctuations in amyloidogenic propensity, despite its low value in the *C. albicans* proteins. This finding corresponds to its low sequence conservation (Figure 4). Overall, the ^69^TSYVGV^374^ and ^322^SNGIVIVATTRTV^334^ segments in the Als5 T domain appear to have the most conserved amyloidogenic propensity among examined segments, at least across close homologs of *C. albicans*.

### Amyloidogenic Propensity Within the Als5 Ig-Like/Invasin and T Domains in Eight Species

**FIGURE 5:**
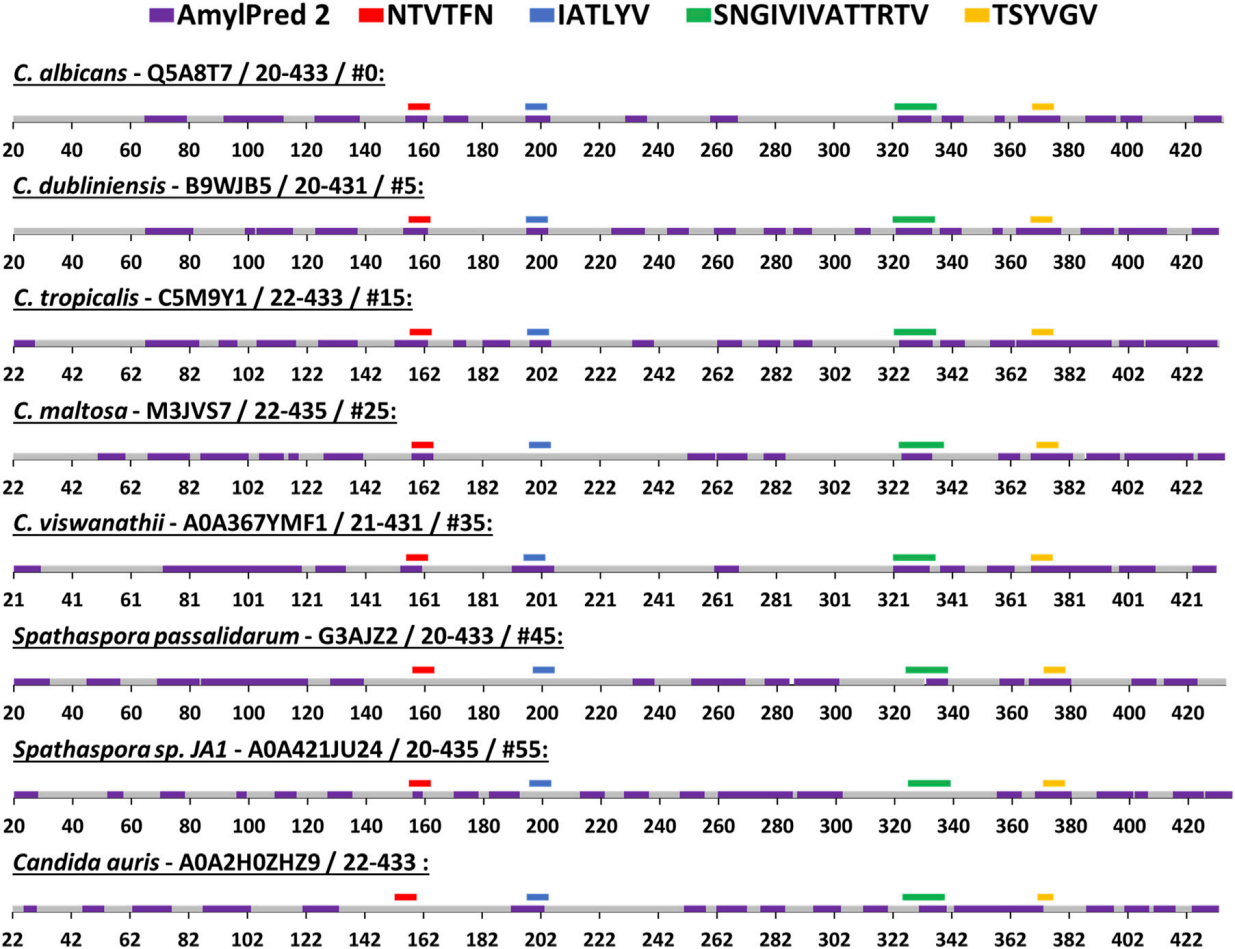
Predicted segments with amyloidogenic propensities in the Als5 Ig-like/invasin and T regions of *C. albicans* and seven orthologs. The amyloidogenic propensity, calculated using the AmylPred2 server (Tsolis et al., 2013), of Als5 Ig-like/invasin and T domains of *C. albicans* and seven orthologs. These specific orthologs were chosen as a diverse group among the 71 homologs collected by the ConSurf webserver along with *C. auris*, as described in the Method section. Each ortholog is identified by the name of the species, their Swissprot ID, residue range of the Ig-like/invasin and T regions and their similarity to *C. albicans* ranked among 71 homologs (determined using the ConSurf-generated multiple sequence alignment and marked with #). The regions with predicted amyloidogenic propensity are marked purple on the sequences, represented as a linear stretch with residue position marks. Regions equivalent to the *C. albicans* sequences are marked above the sequence with different colors as indicated: ^156^NTVTFN^161^ (red), ^196^IATLYV^201^ (blue), and ^369^TSYVGV^374^ (yellow) and ^322^SNGIVIVATTRTV^334^ (green).

We compared the amyloidogenic propensity of the entire Als5 Ig-like/invasin and T domains in Als5 and homologs from seven species: *Candida dubliniensis*, *C. tropicalis*, *C. maltosa*, *C. viswanathii*, *Spathaspora passalidarum*, *Spathaspora* sp. *JA1*, and *C. auris* (Muñoz et al., 2021). The amyloidogenic propensities predicted by AmylPred2 are summarized in Figure 5, with amyloidogenic sequences marked in purple. Each of the adhesins was predicted to have amyloid-forming sequence at the equivalent region to the ^322^SNGIVIVATTRTV^334^ segment of *C. albicans* Als5 (marked green), except for one homolog from *Spathaspora* sp. *JA1*. The equivalent region to the LARKS-like segment ^369^TSYVGV^374^ also showed a strong amyloidogenic propensity (marked dark yellow). In the *C. auris* homolog, there was no equivalent sequence to ^369^TSYVGV^374^, but other segments in this region (residues 343–370) were predicted to be amyloidogenic (marked purple). In the Ig-like/invasin region, the equivalent sequence to ^196^IATLYV^201^ (marked blue in Figure 5) showed no amyloidogenic propensity in *C. maltosa* and *S. passalidarum* or *Spathaspora* sp. *JA1*, but this segment did show some amyloidogenic propensity in *C. auris*. The predicted amyloidogenic propensity of this region was the least conserved among the distant homologs examined in Figure 4B. The segment equivalent to ^156^NTVTFN^161^ showed amyloidogenic propensity predictions (marked red in Figure 5) across the homologs except for *S. passalidarum* and *C. auris*. Overall, each ortholog shown in Figure 5 contains various regions with strong prediction for amyloidogenic propensity. Among the identified segments of Als5, the T-domain segments displayed more conserved amyloidogenic traits compared to segment in the Ig-like/invasin region.

### Multimerization of Als5

**FIGURE 6:**
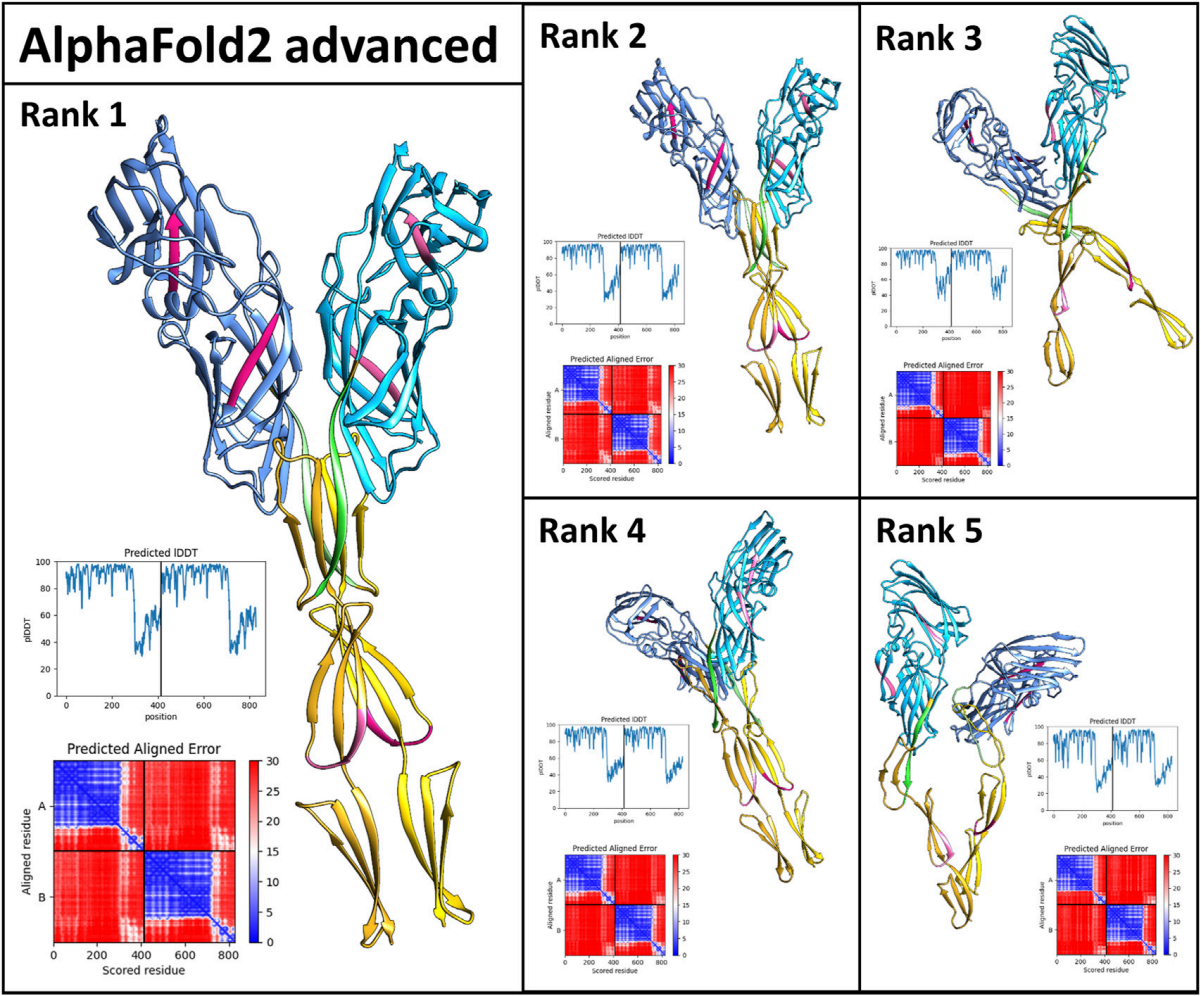
AlphaFold2_Advanced models of Als5 dimers. The model is based on the sequences of the Ig/T domains, colored as in Figure 1. The ranking is based on AlphaFold confidence score. The scores are quantified by the graphs showing the confidence score lDDT plotted against the sequence and the alignment plots (Mariani et al., 2013).

Als5 polymerizes in solution to form initial oligomers which convert to SDS-resistant oligomers, and then to amyloid fibers (Otoo et al., 2008). To gain insight into these interactions, we used AlphaFoldv2 Advanced (modification of AlphaFold v2.1.0 Multimer pipeline) to model the formation of Als5 dimers in the Ig-like/invasin and TR regions (Jumper et al., 2021) to model interactions between two copies of the Ig-like/invasin and T domains Als5. The model suggested dimerization of the folded monomers with an interface roughly parallel to long axis of the protein (Figure 6). In the three highest-ranked models, the major interface was mediated by the T domains, with minor contacts between the Ig-like/invasin domains. The highest-ranked model had the most extensive interface. The predicted amyloidogenic segments are not a part of the interface, but those in the T domain are close to their equivalent segment in the facing monomer. The models are based on the folded structure of the Als5, and do not show partial misfolding into an amyloid state. It is possible that future development of AlphaFold and other methods will allow modeling the cross-β fibrils.

## Discussion

The structures and analyses presented here support the predictions of functional amyloid formation for fungal adhesins. Of the segments identified as potentially forming amyloids, we argue on the bases of structural and evolutionary constraints that two of them in the T domain are likely to function *in vivo*. We note that Als5 might have main and minor aggregation sites, with the latter taking over in case of interference or mutation in the main site, as previously suggested for Tau involved in Alzheimer’s disease and for Fap amyloids involved in the biofilm of *Pseudomonas* (Christensen et al., 2019; Annadurai et al., 2022). We also showed atomic-level cross-β spine structures with known and novel geometries, including one LARKS-like structure reminiscent of functional and reversible human amyloids (Guenther et al., 2018; Hughes et al., 2018).

### Amyloid Predictions

Sequence- and structure-based amyloid predictors generally identify amyloidogenic or β-aggregation potential in many protein sequences. Indeed, systematic studies show that the majority of small peptides with high potential amyloidogenic sequences do form amyloids *in vitro* (Porat et al., 2003; Eisenberg and Sawaya, 2017; Hughes et al., 2018; Bera et al., 2019)*.* However, only few of the proteins containing these sequences form amyloids *in vivo*, probably because peptides are constrained by being part of a stable protein fold *in situ*. Formation of cross-β structures depends on conformation, solvent accessibility, and compatible geometry in flanking regions (Shewmaker et al., 2011; Eisenberg and Sawaya, 2017; Bera et al., 2019). Therefore, amyloids can often form only after proteins denature to expose the amyloidogenic sequences and allow flexibility in the peptide chains. In Als5, we identified 9 segments with high potential to form amyloid-like cross-β structures. We argue here two of these sequences are involved in functional amyloid formation, while 6 others probably do not form cross-β structures *in situ* in the protein. The arguments are based on structural and evolutionary arguments, and on positive evidence for functionality of one segment.

### Potential Amyloidogenic Sequences in the Ig-Like/Invasin Domain

Of the six potential amyloidogenic β-aggregating sequences in the Ig-like/invasin domain of Als5, three are within disulfide-stapled regions of the protein and so these segments are unlikely to be solvent-exposed and flexible enough to form cross-β structures (Figure 1). Of the three potential amyloidogenic sequences between that are not disulfide-constrained, ^156^NTVFN^160^ and ^196^IATLYV^201^ formed cross-β spines *in vitro*. However, these segments are unlikely to do so *in vivo.* Although the sequence ^156^NTVTFN^161^ is relatively highly evolutionary conserved (Figure 4A and Supplementary Figure S2), its TANGO amyloidogenic propensity is low and not conserved across homologs. It has greater potential in the other two predictors (Figures 4B, 5). In Ig/invasin subdomain II, ^156^NTVTFN^161^ seals the edge of a β-sheet (Figure 1), and so it contributes to the hydrophobic core of the fold. Therefore, this sequence is expected to be key for the high stability of this region, and this region of the protein unfolds only at high extension forces > 350 pN (Alsteens et al., 2009; Alsteens et al., 2013). In addition, ^156^NTVTFN^161^ is near disulfide-bonded Cys^150^ as well as the domain I/II interface, which both constrain its flexibility and geometry in the native protein. Therefore, this sequence is unlikely to form a cross-β structure *in vivo* in the intact protein*.*

The sequence ^196^IATLYV^201^ is in a β-strand (Figure 1B) and is poorly conserved in sequence (Figure 4A). Its equivalent region is predicted to have amyloidogenic propensity in some of the homologs, but without the specific trend of amyloidogenic propensity conservation along relatively close and distant homologs (Figures 4B, 5). Therefore, it seems unlikely that this segment participates in the formation of functional β-aggregates in Als adhesins. The segment is extremely close to disulfide bonded Cys^204^, and so it is likely to be too geometrically constrained *in vivo* to form cross-β structures.

The four other potential amyloid sequences in the Ig-like/invasin region failed to form cross-β spines. Therefore, there is little support for formation of cross-β structures *in situ* within the Ig/invasin region of the protein.

### Potential Amyloidogenic Sequences in the T Domain

Two of the potential amyloid sequences in the T domain show conservation of their sequence and amyloidogenic propensity. Figures 4B, 5 demonstrate the strong cross-β potential of ^322^SNGIVIVATTRTV^334^ and the conservation of this propensity in this region across homologs. Similarly, the LARKS-like segment ^369^TSYVGV^374^ also shows high conservation of amyloidogenic propensity (Figures 4B, 5) and conservation in sequence as well (Figure 4A and Supplementary Figure S2). Both of these segments showed strong cross-β properties (Figures 2, 3). Also contrasting with the Ig/Invasin region, this region is unconstrained by disulfide bonds and becomes unstructured under low shear stress. Thus, these sequences are likely to be exposed and relatively unconstrained *in vivo* (Alsteens et al., 2009; Alsteens et al., 2010; Lipke et al., 2017)*.* These features are in accord with the known T domain functional amyloid formation.

### Functions of T Domain Amyloid Sequences

The T domain and the sequence ^322^SNGIVIVATTRTV^334^ are essential for many activities of Als5 and its close paralog Als1 (Lipke et al., 2017; Ho et al., 2019). This sequence is necessary for strong cell-to-cell binding, as well as for clustering the adhesins on the cell surface. A single site mutation V326N reduces the TANGO β-aggregation potential by about 20-fold and also reduces aggregation activity in Als5 without affecting ligand binding to the Ig/invasin domain (Garcia et al., 2011). Furthermore, adhesion activity is enhanced in the presence of a homologous peptide, and activity is reduced in the presence of a homologous non-amyloid peptide with the Val to Asn substitution. These are properties expected from a sequence interacting through β-aggregation. The T region unfolds easily under shear, including under physiological shear stresses (Chan and Lipke, 2014; Lipke et al., 2017). Thus, extension forces unfold the T domain, rendering it unstructured giving the conformational flexibility needed for formation of cross-β structures. Accordingly, we have published extensively on the importance of the ^322^SNGIVIVATTRTV^334^ sequence (Garcia et al., 2011; Lipke et al., 2017; Dehullu et al., 2019a). We have also found that the T region is essential for secretion and processing in yeast (Rauceo et al., 2006). This result implies that the deletion affects the entire fold of the domain.

The discovery of the highly-conserved T-region ^369^TSYVGV^374^ segment that can form a cross-β structure explains several observations. Specifically, Als5 and Als1 show residual aggregation activity and thioflavin binding after mutations that abolish the amyloid potential of the major T domain sequence ^322^SNGIVIVATTRTV^334^ (Alsteens et al., 2010; Garcia et al., 2011; Chan and Lipke, 2014; Dehullu et al., 2019a; Dehullu et al., 2019b; Ho et al., 2019). Similarly, there is 25%–40% residual activity after treatment with a specific peptide that inhibits cross-β formation of the ^322^SNGIVIVATTRTV^334^ segment (Garcia et al., 2011; Alsteens et al., 2013; Chan and Lipke, 2014; Dehullu et al., 2019a; Dehullu et al., 2019b). The residual activity has characteristics of cross-β structures because the cell surface remains somewhat thioflavin T fluorescent. Also consistent with cross-β functional bonds, the activity can be inhibited by concentrations of Congo red or thioflavins 1000-fold higher than those used in fluorescence. At these concentrations, these dyes are sequence-independent inhibitors of amyloid formation (Garcia et al., 2011; Landau et al., 2011; Harris, 2012; Chan and Lipke, 2014). These observations support the idea that another cross-β forming sequence mediates the residual activity. In general, LARKS-like sequences generate transient, low energy cross-β aggregates (Guenther et al., 2018; Hughes et al., 2021). These sequences often mediate reversible liquid-liquid phase separations (Hughes et al., 2018). On the surface of stimulated cells, Als proteins form mobile surface nanodomains visible in atomic force microscopy (AFM) and by *in vivo* staining with thioflavins (Rauceo et al., 2004; Alsteens et al., 2010; Garcia et al., 2011). These nanodomains would be a two-dimensional equivalent of such reversible liquid-like associations.

Therefore, evolutionary conservation, the presence of liquid-like surface nanodomains, the residual amyloid-like behavior in fluorescence and AFM are consistent with the LARKS-like sequence ^369^TSYVGV^374^ providing a supportive role in adherence. Thus, the use of our screens for cross-β sequences has resulted in discovery of a new cross-β segment that appears to contribute to the activity of amyloid-like sequences in the T domain of *Candida* Als adhesins.

*In vivo*, the T domain is unfolded by flow-induced shear stress over mucosal surfaces as low as 0.1–10 (dyne/cm^2^) (Chan and Lipke, 2014). These values are well-within the range of shear stresses in mucosal flow and in the blood stream. Shear-triggered unfolding could thus lead to peptide extension and exposure of segments with cross-β potential, and then to the formation of cross-β aggregates *in vivo.* Models for the transition from the well-folded state to cross-β of Als have been recently published (Lipke et al., 2017; Lipke et al., 2021).

## Conclusion

Atomic resolution structures show that sequences in Als5 can form amyloid-like cross-β aggregates. Of the 4 sequences for which we have structural information, two in the Ig-like/invasin region have characteristics of fortuitous amyloid-formers, i.e., these segments have key roles in a hydrophobic domain core, but they are close to disulfide bonds and one of them has poor conservation of amyloid potential across homologs. In contrast, two sequences in the T domain show characteristics of force-dependent functional amyloids: conservation of sequence and amyloid potential, and the ^322^SNGIVIVATTRTV^334^ segment has demonstrated roles in activity of the protein Als5 and Als1 (Lipke et al., 2017; Ho et al., 2019). The newly discovered LARKS sequence ^369^TSYVGV^374^ is also highly conserved and can explain the residual cross-β activity of Als5 that lacks ^322^SNGIVIVATTRTV^334^ activity. These results demonstrate a role for conservation of amyloid potential as well as sequence in determining functionality of naturally occurring sequences that form β-aggregates. The results illustrate that evolutionary considerations for both sequence and amyloid potential can identify sequences that have high amyloid potential and can potentially distinguish between those that form amyloids *in vivo* and those that do not.

## Data Availability

The datasets presented in this study can be found in online repositories. The coordinates of the atomic structures are available at the PDB *via* the accession codes 6RHA (156-NTVTFN-161), 6RHB (196-IATLYV-201), and 6RHD (369-TSYVGV-374).
