# Supplementary material for: Structure and Conservation of Amyloid Spines From the Candida albicans Als5 Adhesin
Source: Front Mol Biosci. 2022 Jul 6;9:926959. doi: 10.3389/fmolb.2022.926959 (PMC9306254; doi:10.3389/fmolb.2022.926959)
Supplement: Supplementary file 1 [file Presentation1.pdf]

# **Structure and conservation of amyloid spines from the *Candida albicans* Als5 adhesin**

## **Supplemental Table and Figures**

Nimrod Golan[1], Sergei Schwartz-Perov[1], Meytal Landau[1,2]*, and Peter N. Lipke[3]*

[1] Department of Biology, Technion-Israel Institute of Technology, Haifa 3200003, Israel

[2] European Molecular Biology Laboratory (EMBL), and Centre for Structural Systems Biology, Hamburg, Germany

[3] Biology Department, Brooklyn College of the City University of New York, New York, USA

*Correspondence to: PLipke@brooklyn.cuny.edu; mlandau@technion.ac.il

**Table S1. Features of the Als5 spine structures compared to the NNQQNY steric zipper structure**

| | Als5 $^{196}$IATLYV$^{201}$ | Als5 $^{156}$NTVTFN$^{161}$ | Als5 $^{369}$TSYVGV$^{374}$ | Sup35 NNQQNY |
|---|---|---|---|---|
| | 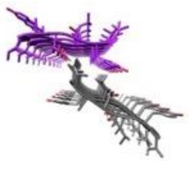 | 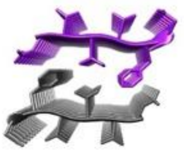 | 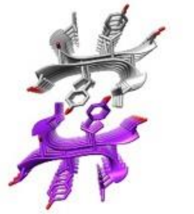 | 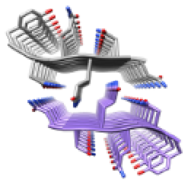 |
| **Shape complem-entarity**[a] | 0.74 | 0.78 | 0.79 | 0.86 |
| **Inter-strand distance along the sheet** | 4.745 Å (9.49 Å between antiparallel strands) | 4.87 Å | 4.68 Å (9.36 Å between antiparallel strands) | 4.87 Å |
| **Area buried of one strand within pairs of sheets**[b] | Strand 1: 563 Å$^2$<br>Strand 2: 606 Å$^2$<br>**Average**: 585 Å$^2$ | 589 Å$^2$ | Strand 1: 581 Å$^2$<br>Strand 2: 401 Å$^2$<br>**Average**: 491 Å$^2$ | 624 Å$^2$ |

The values of shape complementarity, inter-strand distance and solvent exposed surface area buried calculated for the Als5 spine structures are compared with those of the NNQQNY segment from yeast prion Sup35 (PDB code 1YJO)(Sawaya et al., 2007) steric zipper structure. The NNQQNY was chosen for this comparison as it shows one of the highest values of shape complementarity and surface area buried among steric zipper structures(Sammond et al., 2007).

[a]Shape complementarity of 0 indicates no complementarity of the two surfaces and approaches 1 for atomic surfaces that fit perfectly together(Lawrence and Colman, 1993).

[b]The solvent accessible surface area buried is the average area buried of one strand within two β-sheets (total area buried from both side is double the reported number). The surface area buried was calculated with Chimera (UCSF)(Goddard et al., 2007) with default probe radius and vertex density are 1.4 Å and 2.0/Å$^2$, respectively. IATLYV and TSYVGV assemble into antiparallel

β-sheets, hence each of the antiparallel β-strands forms a difference interface that was calculated separately.

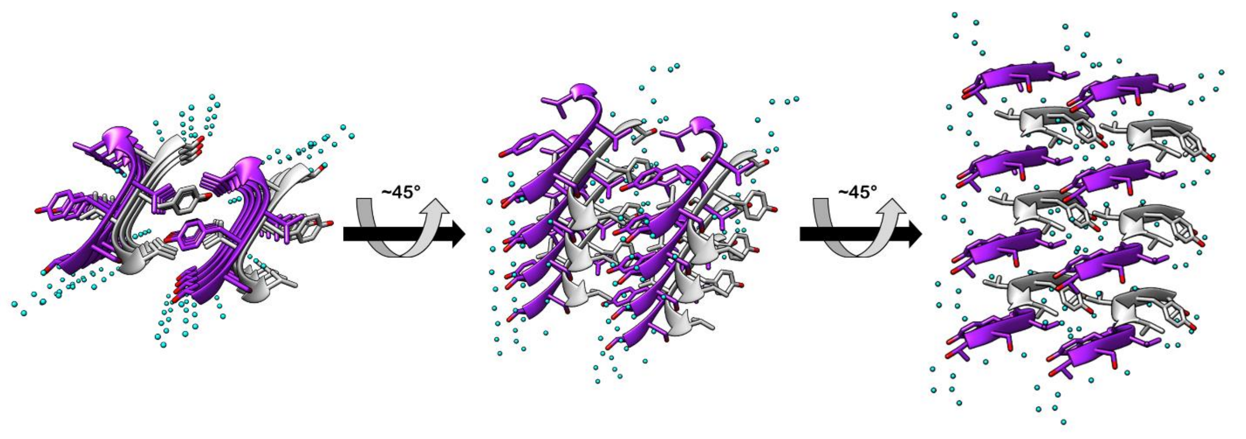


**Supplemental Figure 1. Crystal structures of the T-domain $^{369}$TSYVGV$^{374}$ segments .** The high-resolution crystal structure of $^{369}$TSYVGV$^{374}$ is shown at different angles to better demonstrate its unique LARKS-like structure. The left panel is viewed down the fibril axis, with β-strands shown as ribbons and residues as sticks, while the middle and right panels respectively display a titled view (middle) and a view perpendicular to the fibril axis. The different views demonstrate the unique packed anti-parallel β-sheets composed of twisted strands, similar to LARKS structures of kinked β-strands. β-sheet carbons are colored either gray or purple, and heteroatoms are colored according to their atom type (nitrogen in blue, oxygen in red) and water molecules in cyan.

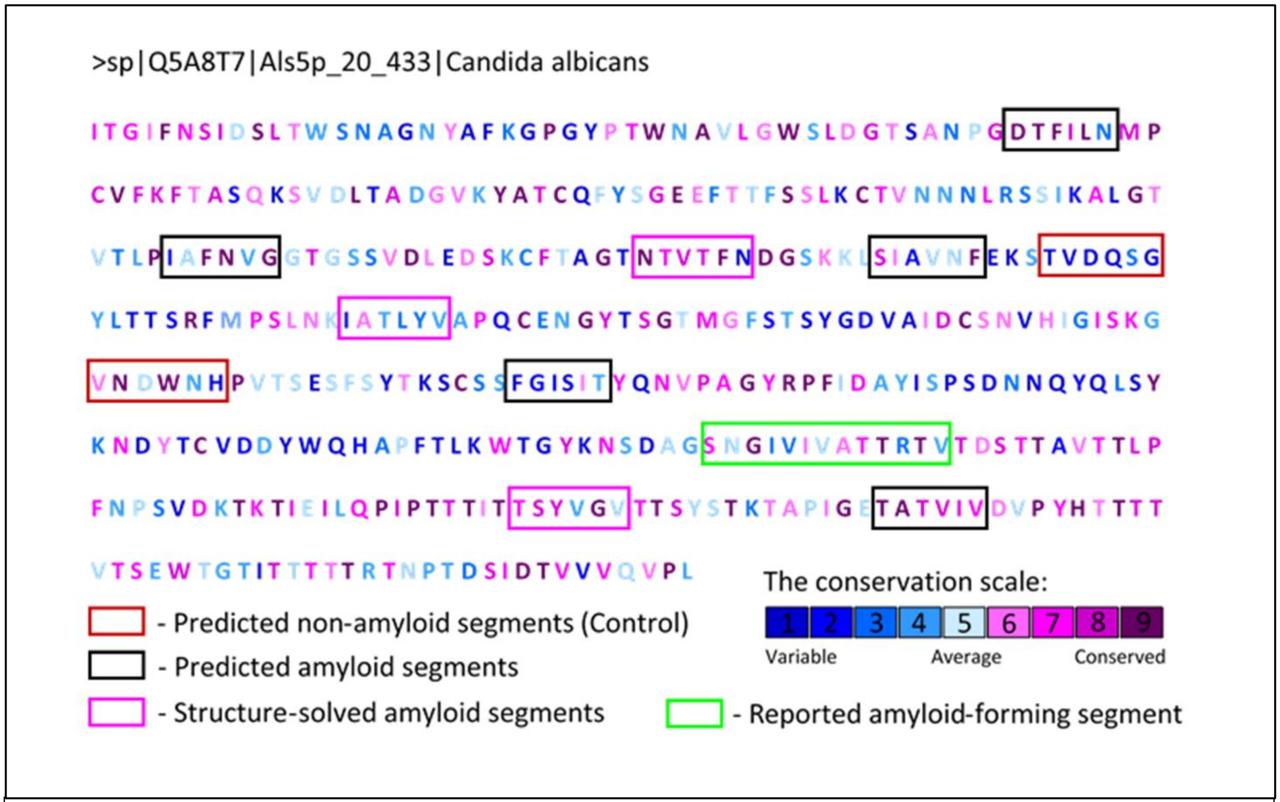


**Supplemental Figure S2. Sequence conservation scores mapped on the Als5(20-433) sequence.**

Evolutionary conservation scores were calculated using the ConSurf webserver (Landau et al., 2005; Ashkenazy et al., 2016; Berezin et al., 2004) based on a multiple sequence alignment and phylogenetic tree of 71 homologous proteins of the of N-terminal region of *Candida albicans* Als5 (UniProt ID Q5A8T7), residues 20-433. The conservation score of each residue is represented by a color scheme ranging from dark blue to dark purple (indicating variable to conserved). Segments for which the crystal structure was determined are marked with magenta boxes, the known amyloid segment $^{322}$SNGIVIVATTRTV$^{334}$ is marked with a bright green box, and predicted amyloidogenic segments which did not crystalize are marked with black boxes. Segments that were not predicted to have amyloidogenic

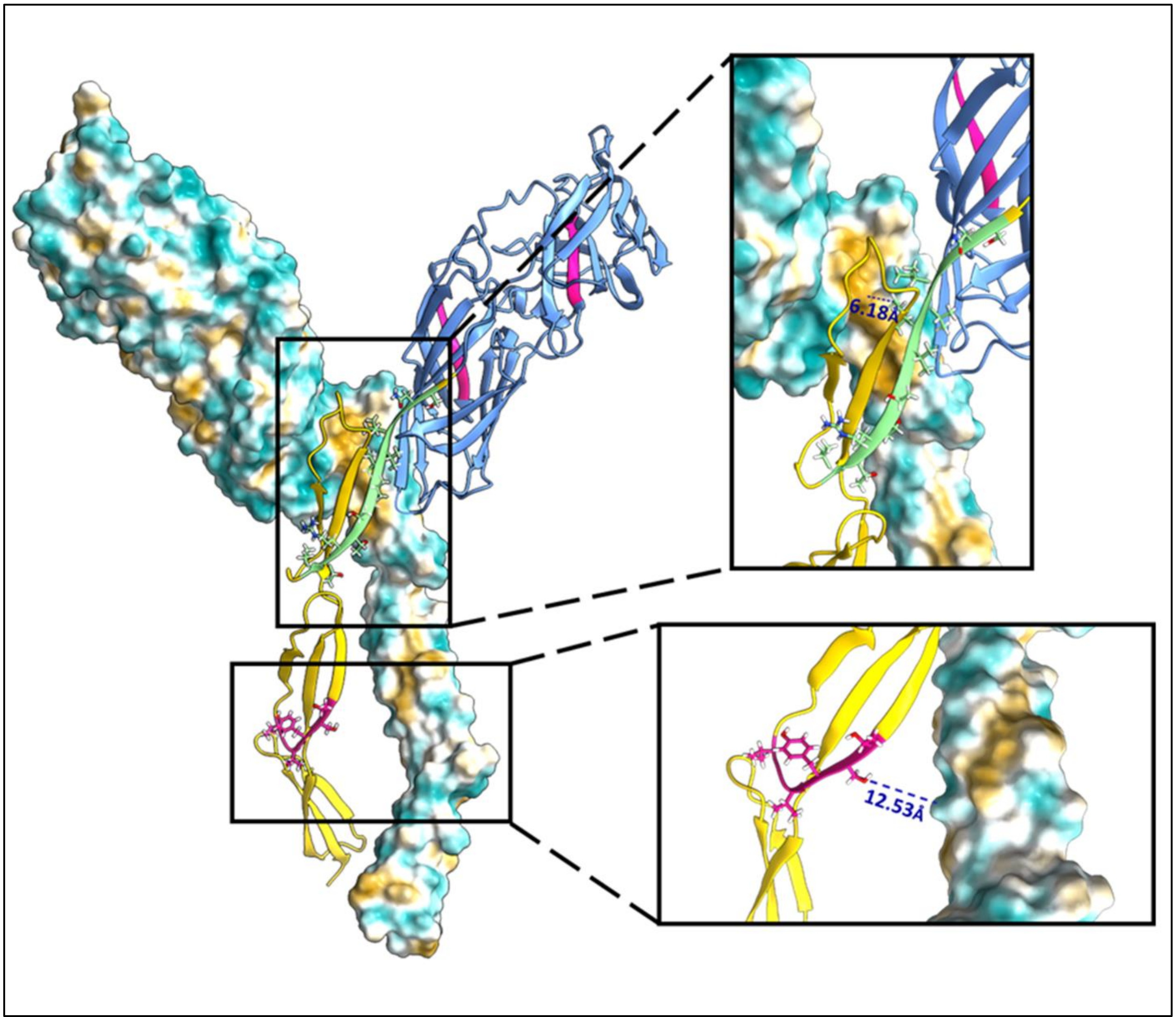


**Supplemental Figure S3.** Quaternary interactions in the top-ranked AlphaFold2 Advanced model of the Als5 dimer. The sequence $^{322}$SNGIVIVATTRTV$^{334}$ is colored green and $^{369}$TSYVGV$^{374}$ is pink. The shortest distance between these segments is marked.

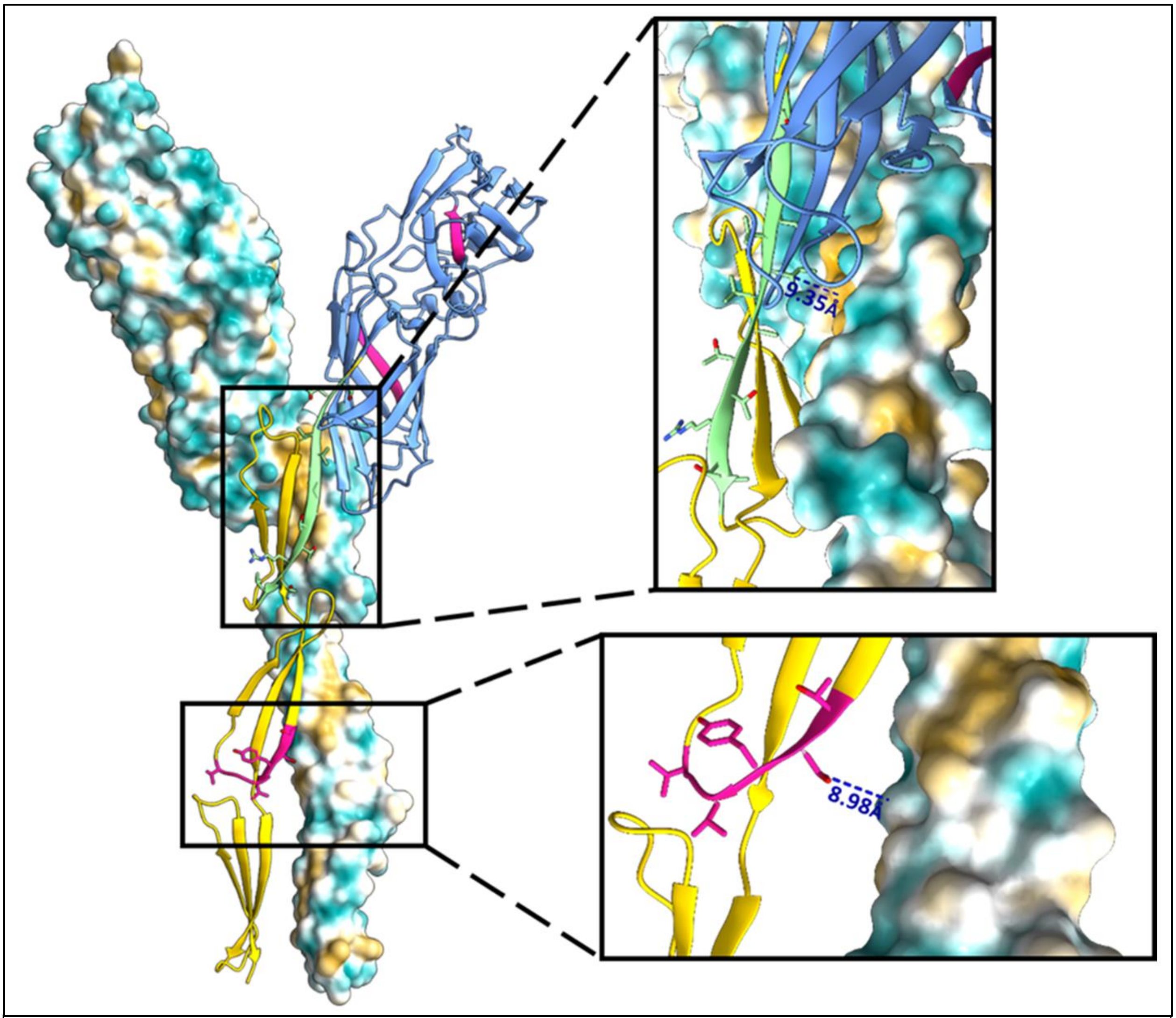


**Supplemental Figure S4.** Quaternary interactions in the second-ranked AlphaFold2 Advanced model of the Als5 dimer. The sequence $^{322}$SNGIVIVATTRTV$^{334}$ is colored green and $^{369}$TSYVGV$^{374}$ is pink. The shortest distance between these segments is marked.

## Literature Cited


Goddard, T. D., Huang, C. C., and Ferrin, T. E. (2007). Visualizing density maps with UCSF Chimera. *J. Struct. Biol.* 157, 281–287. doi: 10.1016/J.JSB.2006.06.010.

Lawrence, M. C., and Colman, P. M. (1993). Shape complementarity at protein/protein interfaces. *J. Mol. Biol.* 234, 946–950. doi: 10.1006/JMBI.1993.1648.

Sammond, D. W., Eletr, Z. M., Purbeck, C., Kimple, R. J., Siderovski, D. P., and Kuhlman, B. (2007). Structure-based protocol for identifying mutations that enhance protein-protein binding affinities. *J. Mol. Biol.* 371, 1392–1404. doi: 10.1016/J.JMB.2007.05.096.

Sawaya, M. R., Sambashivan, S., Nelson, R., Ivanova, M. I., Sievers, S. A., Apostol, M. I., et al. (2007). Atomic structures of amyloid cross-β spines reveal varied steric zippers. *Nat. 2006 4477143* 447, 453–457. doi: 10.1038/nature05695.

Ashkenazy, H., Abadi, S., Martz, E., Chay, O., Mayrose, I., Pupko, T., et al. (2016). ConSurf 2016: an improved methodology to estimate and visualize evolutionary conservation in macromolecules. *Nucleic Acids Res.* 44, W344–W350. doi: 10.1093/NAR/GKW408.

Landau, M., Mayrose, I., Rosenberg, Y., Glaser, F., Martz, E., Pupko, T., et al. (2005). ConSurf 2005: the projection of evolutionary conservation scores of residues on protein structures. *Nucleic Acids Res.* 33. doi: 10.1093/NAR/GKI370.

Berezin, C., Glaser, F., Rosenberg, J., Paz, I., Pupko, T., Fariselli, P., et al. (2004). ConSeq: the identification of functionally and structurally important residues in protein sequences. *Bioinformatics* 20, 1322–1324. doi: 10.1093/BIOINFORMATICS/BTH070.
